# Women drive efforts to highlight concealable stigmatized identities in U.S. academic science and engineering

**DOI:** 10.1371/journal.pone.0287795

**Published:** 2023-07-19

**Authors:** Carly A. Busch, Katelyn M. Cooper, Sara E. Brownell

**Affiliations:** Research for Inclusive STEM Education Center, School of Life Sciences, Arizona State University, Tempe, Arizona, United States of America; G. D’Annunzio University of Chieti-Pescara, ITALY

## Abstract

Concealable stigmatized identities (CSIs) are hidden identities that carry negative stereotypes and can result in a loss of social status if revealed. Instructors often choose to conceal these CSIs due to anticipated negative student reactions, yet revealing CSIs can have a positive impact on undergraduates. Women are generally more likely to reveal personal aspects about themselves in social situations, but may face greater consequences for revealing a stigmatized identity to students given their already marginalized position in academic science and engineering. Therefore, in this study, we were interested in understanding to what extent there are differences between men and women science and engineering instructors in (i) the representation of CSIs, (ii) their decisions to reveal CSIs to undergraduates in their classes, and (iii) their perceived stigma of CSIs. Based on a national survey of over 2,000 instructors in science and engineering from very high research activity doctoral institutions, we found that women were more likely than men to report having depression, anxiety, or a disability. Of instructors who held CSIs, women had 1.5x higher odds than men of revealing their CSIs to some undergraduates compared to no undergraduates and perceived greater stigma associated with all CSIs. Despite perceiving greater stigma associated with concealable stigmatized identities, women are more likely to reveal their CSIs to college science and engineering students, leading the way to a more diverse and inclusive scientific community by demonstrating themselves as role models for these identities.

## Introduction

### Science and engineering environments are devoid of identity sharing

Science and engineering (S&E) instructors and classrooms are notoriously chilly and unapproachable [1–3]. The assumed objectivity of S&E often results in instructors leaving any part of their personal identity outside of the classroom, which results in students not seeing the humanizing aspects of the instructor. Therefore, there are an increasing number of calls to make S&E departments and learning environments more welcoming to individuals from a variety of backgrounds and identity groups [4–7]. However, if the overall culture of S&E remains unchanged, instructors may be reluctant to share personal identities with students due to established norms.

### Instructor identities

Some identities, such as gender and ethnicity, are generally considered to be visible such that others know, or assume, an individual holds a particular identity without being told [8]. However, while gender is typically assumed to be a visible identity, that may not be the case for everyone, particularly for genderqueer and non-binary individuals [9,10]. Other identities are widely considered to be concealable, such as having depression, low socioeconomic status, and LGBTQ+ identities [11,12]. The combination of identities an individual has may lead to intersectional inequalities or privileges. For example, white able-bodied cisgender heterosexual men experience more privileges than individuals with other combinations of gender, race, sexual identity, and disability status [13]. Therefore, it is important to explore how individuals, including instructors, navigate academic S&E spaces differently based on their own combination of visible and concealable identities.

### Instructor concealable stigmatized identity disclosure

Some instructors of college S&E courses aim to be approachable to their students to improve student outcomes in the course [1,14,15]. Sharing personal experiences and information including hobbies, pets, family, and identities or characteristics can help foster positive relationships with undergraduates [16–20]. Although limited, some recent research suggests that sharing concealable stigmatized identities (CSIs) may also help build student-instructor relationships and disproportionately so for students who share these marginalized identities [16]. Concealable stigmatized identities (CSIs) are identities which can be hidden and carry negative stereotypes when revealed depending on the context [12,21,22]. In the United States, concealable stigmatized identities generally include LGBTQ+ identities, having depression or anxiety currently or in the past, and low socioeconomic status [11,23]. Although U.S. academic S&E may differ in its cultural values from the U.S. at-large (i.e., the U.S. remains primarily Christian in its values whereas S&E tend to be more secular spaces [24–26]), many of these identities are stigmatized in both contexts [11,23,27–31].

Undergraduates with CSIs who feel marginalized in science anticipate that science faculty revealing the same CSI could provide them with a role model to relate to based on their shared identity [28,32]. Additionally, instructors have described that revealing their LGBTQ+ identities to students can benefit students by making LGBTQ+ students feel more comfortable in the classroom and by modeling authenticity [33,34]. Indeed, when an instructor revealed a CSI to undergraduates during class, students largely perceived a positive impact on their course experience, especially the students who shared the CSI [16,35].

Owing to the benefits thought to be associated with revealing CSIs, instructors are often cautiously encouraged to consider sharing these details if they are comfortable. However, the extant literature highlights reasons why instructors may be reluctant to share this information with students, even when students could benefit. S&E instructors may keep identities concealed because they perceive that disclosing an identity is inappropriate [28,36,37] or are concerned about students’ negative perceptions of individuals with the identity [23,38,39].

The decision of whether to reveal a CSI to students can be compounded with other challenges individuals face in the S&E workplace. Specifically, disadvantaged groups in S&E (e.g., women) may choose to not reveal a CSI to avoid adding more stigma to themselves. Despite the associated stigma, an instructor may choose to reveal a CSI to be an example to students of a successful scientist with that identity [26,33] or because they prefer to live authentically [23,33]. Characteristics associated with certain identity groups may also influence whether instructors choose to reveal CSIs. Specifically, women are generally more empathetic than men [40] and thus may be more perceptive to stigma. Consequently, men and women may differ in the extent to which they disclose CSIs to undergraduates because the degree of perceived stigma affects individuals’ decisions to reveal CSIs [38,39].

### Gendered expectations for instructors in science and engineering

Students’ expectations for instructors and mentors in S&E can vary based on the instructor’s gender. While gender exists beyond the binary of man and woman [4,41], previous literature and the current study focus on these two gender identities. Men often score higher on student evaluations of teaching [42,43], but student evaluations of teaching have been criticized for measuring conformity with gendered expectations rather than teaching quality. For example, women were often lauded on evaluations for being “supportive” and criticized for not spending enough time and emotional labor outside of class [44]. Additionally, women report providing undergraduate and graduate student researchers more psychosocial support than men, whereas men report providing more career development than women [45]. Students may perceive women and men differently based on gendered expectations, which may influence instructors’ behavior including whether they reveal CSIs to their students. Specifically, students’ gendered expectations may lead women to reveal CSIs more than men.

### Current study

The ways in which instructors navigate academic science and engineering with concealable stigmatized identities is likely affected by gendered experiences and expectations. We explored eight CSIs in the context of men and women faculty in S&E: lesbian, gay, bisexual, and queer (LGBQ+) identities [46], depression [28], anxiety [47], struggling academically [48], growing up in a low income household [49], transferring from a 2-year institution [50], being a first-generation college student [51], and having a disability [52]. These eight CSIs were chosen because they all represent identities that can be concealed, so one would need to reveal the identity. Additionally, there is an associated stigma with each identity that could be considered marginalized in society and in academic S&E spaces.

Therefore, we sought to explore to what extent there are gender differences between men and women science and engineering instructors in (i) reporting CSIs, (ii) revealing CSIs, and (iii) rating the stigma associated with CSIs. Revealing CSIs to undergraduates may be a way to both foster connections with students and provide role models of successful scientists with these stigmatized identities, so understanding the extent to which women and men are assuming this responsibility and any gender differences will further illustrate the ways that gender affects instructors’ experiences in science and engineering.

## Methods

This study was conducted under an approved Institutional Review Board protocol from Arizona State University (#00013208). Written consent was obtained from all participants.

### Survey design and validation

We developed a survey with closed-ended items to assess whether science and engineering faculty and instructors hold identities that are often considered to be concealable and stigmatized in the broader United States and whether they reveal or conceal those identities to undergraduate students. We conducted 6 cognitive think-aloud interviews with undergraduate science and engineering instructors to establish cognitive validity and ensure that items were being interpreted as intended [53]. The survey items were iteratively revised after each think-aloud. A copy of the survey questions analyzed is provided in the **S1 Appendix**.

#### Screening questions

The survey began with questions regarding the instructor and the undergraduate course they teach most often. Participants who did not teach undergraduate students were sent to the end of the survey and were not included in the study. Participants provided information about the course including course subject, size, and most recent time teaching. Those who do not teach science (defined as biology, geosciences, chemistry, and physics) or engineering were sent to the end of the survey and not included in the study.

#### Identities of interest

All identities of interest in the survey had evidence of being concealable and stigmatized in the U.S. and in the context of science and/or academia. Specifically, participants were asked whether they identify as lesbian or gay, bisexual, having anxiety, having depression, being a first-generation college student, growing up in a low-income household, struggling academically in college, being a community college transfer student, or having a disability. We selected these eight identities based on the prior literature documenting concealable stigmatized identities in STEM workplaces and U.S. contexts. Because individuals can hold identities that are stigmatized only in specific contexts (e.g., there is evidence that Christians are stigmatized in science but not the U.S. broadly [26]), we included only identities which had evidence of stigma across both contexts.

Below we detail the eight identities we investigated, which are considered to be concealable stigmatized identities in the context of both the U.S. at-large as well as in academic science and engineering (**Table 1**). Lesbian, gay, bisexual, and queer (LGBQ+) individuals face discrimination and bias in S&E [46], and LGBQ+ undergraduates are underrepresented in the sciences [54]. Concealable stigmatized identities associated with mental health, namely depression and anxiety, affect undergraduates’ experiences across learning environments [47,55–62].

**Table 1:** Rationale for including each of the eight concealable stigmatized identities, including evidence of discrimination or attrition and disadvantage or stigma for each.

Identity	Discrimination/attrition	Disadvantage/stigma
LGBQ+ identities	LGBQ+ undergraduates are underrepresented in the sciences [54].	Homophobia and heteronormative biases are prevalent throughout S&E [46,63].
Depression	Depression is associated with lower academic performance, including GPA [64,65].	Undergraduates often conceal depression due to anticipated stigma [28,66].
Anxiety	Anxiety levels are inversely related to persistence in a biology major [47,60].	STEM environments can contribute to worsening students’ mental health, including anxiety [67,68] and undergraduate science learning environments can exacerbate fear of negative evaluation, an underlying cause of student anxiety [57,59].
Struggle academically	Poor academic performance, including GPA, is associated with lower persistence in STEM majors [48,69].	Students often cite problems with grades as a primary reason for leaving STEM majors [2].
Low income growing up	Students from low SES backgrounds are less likely to pursue a STEM major [49].	Students from low SES backgrounds feel a disconnect between their college environment and where they grew up [29] and may be unable to pursue unpaid undergraduate research opportunities [70].
Community college transfer	Relatively few students enrolled in community college STEM courses progress to advanced STEM coursework, including transferring to 4-year institutions [71].	Students who transfer from community colleges to 4-year institutions often experience transfer shock, which is more pronounced in science majors [72].
First- generation	First-generation college students are less likely to persist in STEM majors [51].	First-generation students have to navigate college without parental guidance [73] and face a cultural mismatch upon entering college [74].
Has a disability	People with disabilities are underrepresented in STEM professions [31].	Learning environments and research experiences often lack necessary accommodations [52,75] and students often face negative attitudes from faculty and peers due to their disability [31].

Related to the undergraduate experience, students who struggle academically [2,48,69,76], grew up in a low income household [29,49], transferred from a community college or other 2-year institution [50], or are a first-generation college student [51,74,77] may perceive stigma and/or bias due to those characteristics. Additionally, individuals who have a disability face many challenges in S&E [31,52,78] and individuals with hidden disabilities often face the additional challenge of needing to disclose their disability to receive the accommodations that they need [75,79].

#### Extent of reveal

For each identity of interest, faculty participants with the identity were asked to consider the last time they taught the course they indicated earlier on the survey and whether they revealed their identity to all undergraduates enrolled in the course, to some undergraduates enrolled in the course (e.g., during office hours), or if they did not reveal the identity to undergraduates in the course. We chose to focus on the extent to which participants revealed their identity to undergraduate specifically because undergraduates who have at least one of the identities of interest have higher attrition from S&E programs than their counterparts [31,47–49,51,54,60,64,65,69,71,80] and examples of diverse and/or counter-stereotypical examples of scientists can improve undergraduate outcomes such as self-efficacy and academic achievement [81–84]. Instructors revealing these identities to graduate students instead of undergraduates may be too late in the training process to have meaningful effects on diversifying who stays in S&E. Additionally, instructors have described revealing LGBTQ+ identities more often in upper level courses with small sizes compared to larger lower level courses [34], which may extend to other CSIs and lead to instructors in general being less likely to reveal CSIs to undergraduates compared to graduate students.

#### Stigma ratings

To assess the level of stigma associated with each identity, we provided a definition of stigmatized (“To be stigmatized means to be culturally devalued, prejudiced, or negatively stereotyped due to a particular identity and is influenced by the culture of a particular context”) and then participants responded to the question, “To what extent do you perceive that identifying as [identity of interest] is stigmatized in the context of academic science and engineering?” Participants responded on a four-point scale from “not stigmatized” (1) to “extremely stigmatized” (4) with the option to select “I do not know what this identity is.” Although each identity included in the survey had prior evidence of stigma in academic science contexts, we asked participants about their perception of stigma against the identities of interest to ensure that the sample considered them to be stigmatized and because we hypothesized that an individual’s identities would affect the extent to which they perceived stigma. All participants ranked their perceived stigma for all identities. We asked about each identity individually and converted the text-based responses to their numerical counterparts. For a stigma rating for LGBQ+ identities, we averaged participants’ rankings for gay or lesbian and bisexual identities.

#### Additional demographic information

We collected faculty participant demographic information including appointment type, race/ethnicity, gender, and age in addition to the identities of interest.

### Survey distribution

We identified faculty and instructors across science and engineering departments from all very high-research doctoral granting institutions in the USA via publicly available websites and collected their names and email addresses. We recruited participants (n ≅ 50,000) via email using a mail merge service. Participation was incentivized by awarding the first 50 individuals who completed the survey a $100 gift card and entering all participants into a drawing for one of two $500 cash awards. We sent the initial invitation to participate in the study in November 2021 and a final reminder in January 2022.

### Data analysis

We calculated the percent of participants by binary gender who reported each of the identities of interest. While gender exists on a spectrum beyond the binary of man and woman [4,41], individuals whose gender identity falls outside of this binary also face discrimination in S&E often rooted in both sexism and homophobia [80]. Therefore, we only included men and women in order to exclude any influence of homophobia or bias against non-binary or genderqueer individuals from our interpretations. To assess whether there were gender differences in reporting each identity of interest, we used logistic regression analyses, controlling for race/ethnicity, age, and appointment type. (Model: has identity (0/1) ~ gender + race/ethnicity + age + appointment).

We calculated the percent of participants by binary gender who revealed each identity to all, some, or none of their undergraduate students; we also calculated these percentages for all CSIs combined. To assess whether there were gender differences in the extent to which instructors reveal their CSIs, we used multinomial logistic regression analyses. We again included race/ethnicity, age, and appointment as predictors as well as included individual as a random effect to account for participants with multiple CSIs. (Model: extent out (all/some/none) ~ gender + race/ethnicity + age + appointment + (1|individual)).

For stigma ratings, we calculated the percent of participants by binary gender who selected each option. To assess whether there were gender differences in the stigma rating participants assigned to each identity, we used ordinal regression analyses with the same predictors as described previously. (Model: stigma rating (not at all/little/somewhat/extremely) ~ gender + race/ethnicity + age + appointment + (1|individual).) We assessed global differences in stigma ratings by calculating the overall percent of participants by binary gender who selected each option regardless of identity being rated. We used the same ordinal regression described above to identify overall differences in stigma ratings.

All analyses were completed in R [85] using the stats (logistic) [85], nnet (multinomial) [86], and ordinal (ordinal) [87] packages for each of the three types of regression analyses. For all binary and multinomial logistic regression analyses, we confirmed there were no outliers and all assumptions were met. For all ordinal regression analyses, proportional odds assumptions were checked and met. For all models, multicollinearity among predictors was assessed via the variance inflation factor (VIF) values using the car package [88]. The VIF values indicated no issues with multicollinearity. Throughout the manuscript we specify significant results at the threshold of p ≤ .05, and the full results of all regression models are included in the **Supporting Information**. The code can be found online (https://github.com/carlybusch/Women-highlight-CSIs-in-science.git) and de-identified data in the **S2 Appendix**.

### Positionality statement

All three authors identify as women in biology; two of the authors are faculty members and routinely reveal at least one CSI that they hold to all undergraduates in the courses that they teach.

## Results

In total, 2,013 instructors, tenure-track, and tenured science and engineering faculty members who teach undergraduates participated in the survey. 38% of the participants were women, 58% were men, 0.5% were non-binary or genderqueer, and 3% declined to state their gender. Individuals who declined to state their gender or who identified as non-binary or genderqueer were excluded from the analyses. Participants represented a range of ages and were primarily white (**Table 2**).

**Table 2:** Participant demographic information.

Demographic		Percent (n)
Age	23–37	19.8 (398)
	38–49	33.6 (676)
	50–59	18.7 (377)
	60+	19.0 (383)
	Decline to state	8.9 (179)
Race/ ethnicity	White	72.1 (1451)
	Asian	13.0 (262)
	Hispanic, Latino/A, Or Of Spanish Origin	4.5 (90)
	Other (Including Multiracial)	2.8 (57)
	Decline To State	5.6 (113)
	Black Or African American	1.6 (32)
	American Indian Or Alaska Native	0.3 (5)
	Pacific Islander	0.1 (2)
	Native Hawaiian	0.1 (1)

### Women are more likely than men to report having depression, anxiety, or a disability

**Fig 1:**
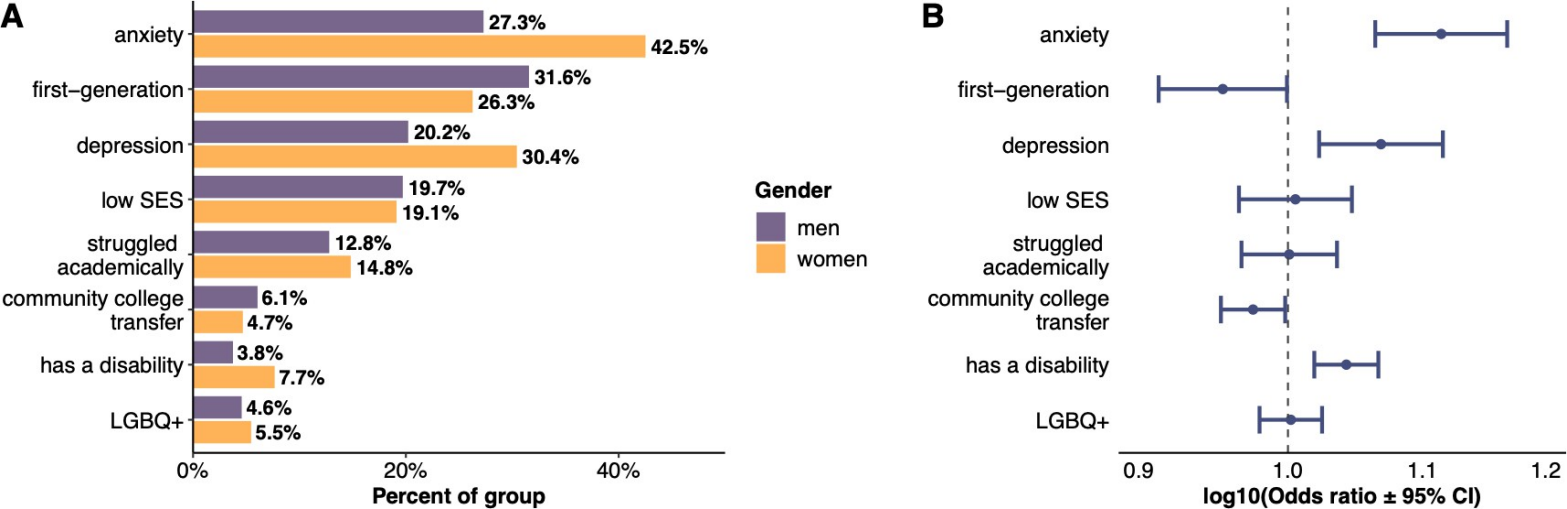
Gender differences between men and women in reporting concealable stigmatized identities. **A)** Percent of men (purple) and women (yellow) who reported having each concealable stigmatized identity. **B)** Logistic regression results demonstrate that women are more likely to report having depression, anxiety, or a disability but less likely to report being a first-generation college student or transferred from a community college. Points to the right of the vertical dashed line indicate women are more likely than men to report the identity; confidence intervals which do *not* cross the line are statistically significant.

A higher percentage of women than men report having anxiety, depression, having struggled academically in college, having a disability, or being LGBQ+ (**Fig 1A**; full demographic information available in **S1 Table**), with the largest differences in percent for reporting depression or anxiety. To better assess whether these differences may be specific to gender, we conducted a logistic regression controlling for other factors hypothesized to explain such differences, namely race, age, and appointment [89,90]. When race, age, and appointment are accounted for, women are more likely than men to report having depression (OR = 1.08, p < .001), anxiety (OR = 1.12, p < .001), and having a disability (OR = 1.04, p < .001) but less likely to report being a first-generation college student (OR = 0.95, p = .03) or transferred from a community college (OR = 0.98, p = .04; **Fig 1B**). The full results from the logistic regressions can be found in the **S2 Table**.

### Women reveal concealable identities to undergraduates more readily than men

**Fig 2:**
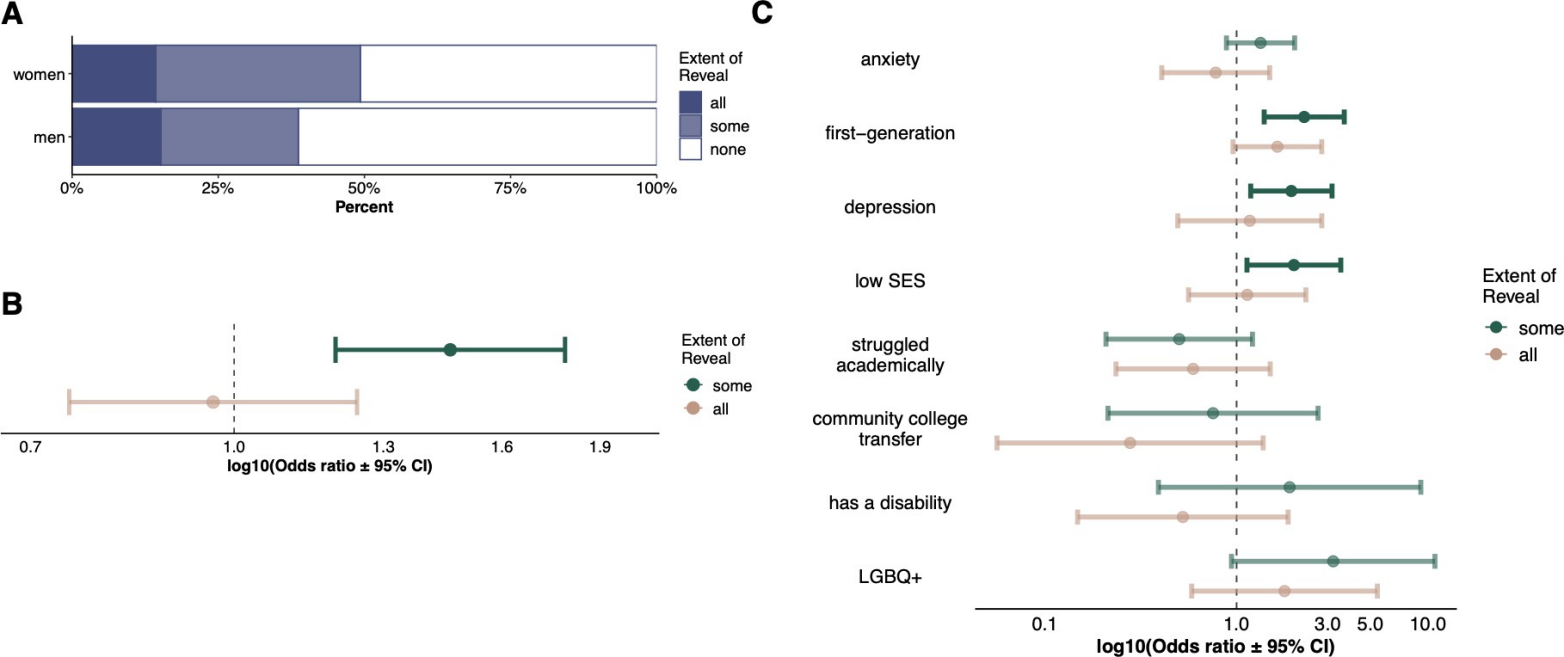
Gender differences between men and women in revealing concealable stigmatized identities. **A)** Percent of men and women who reveal a concealable stigmatized identity (aggregate) to all, some, or none of their undergraduate students. **B)** Women are more likely than men to reveal a CSI to some (compared to none) of their undergraduates; no significant difference between men and women for revealing to all (compared to none) of their undergraduates. **C)** Multinomial logistic regression results for revealing each identity for women compared to men (disaggregated); points to the right of the dashed vertical line indicate women are more likely than men to reveal the identity to some (teal) or all (pink) undergraduates (compared to no undergraduates) and confidence intervals which do not cross the dashed line and are darker are statistically significant.

With regard to whether instructors reveal concealable stigmatized identities to all, some, or none of their undergraduate students, women have 1.46x higher odds than men of revealing CSIs to some students compared to no students (p < .001; **Fig 2A and 2B**). Disaggregating specific identities, women are more likely than men to reveal to *some* students that they have depression (OR = 1.93, p = .008), grew up in a low income household (OR = 1.99, p = .02), or are first-generation college students (OR = 2.25, p < .001) (**Figs 2C** and **S1**). There are no significant differences between men and women in whether they revealed a CSI to *all* students in their classes compared to none of their undergraduates in aggregate or disaggregated by CSI. Full results for all regressions are available in the **S3 Table**.

### Women perceive greater stigma associated with concealable identities than men

**Fig 3:**
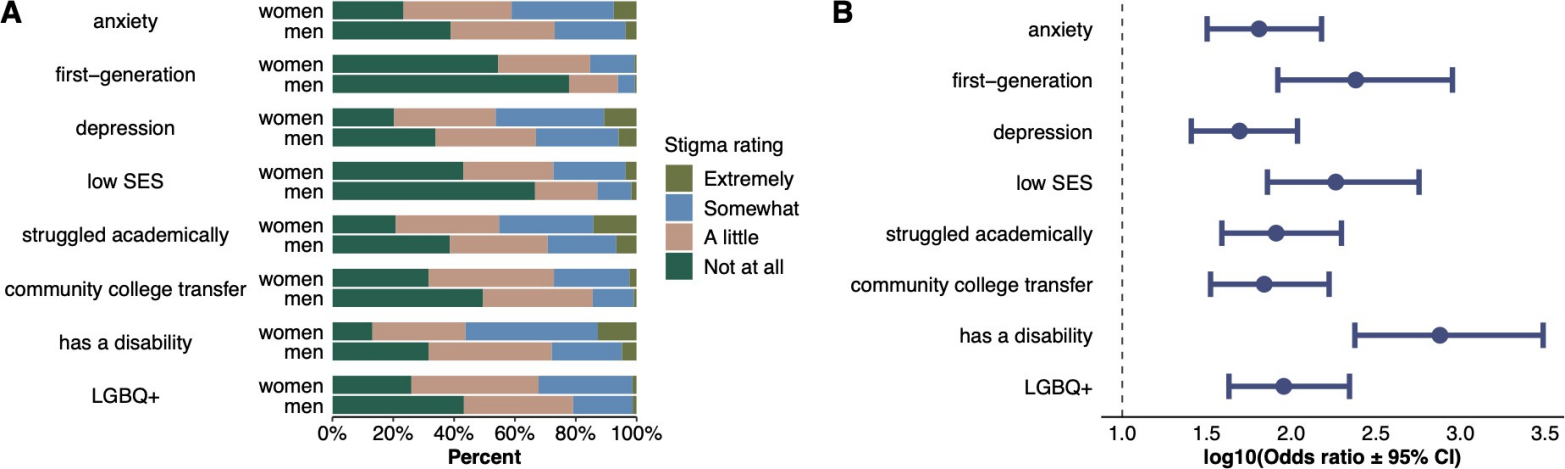
Gender differences between men and women’s perceived stigma for each concealable stigmatized identity A) by percentage and B) based on ordinal regressions. All confidence intervals do not cross the vertical line and are statistically significant; points to the right of the line indicate women rated the stigma associated with the identity higher than men.

Women rate concealable stigmatized identities, in aggregate, as more stigmatized than men (OR = 1.93, p < .001; full result in the **S4 Table**). Further, when disaggregating specific identities, women rate each of the eight CSIs as more stigmatized than men (all p < .001; **Fig 3A and 3B**; full results in the **S4 Table**).

## Discussion

Overall, women disclose CSIs to undergraduates more than men, specifically to *some* undergraduates rather than *all* undergraduates. While this could be due to appointment type–women are more likely to hold non-tenure positions [89]–or age–women are more likely to have been hired more recently and therefore may be younger on average [90]–neither of these factors appear to drive this difference in our dataset. We controlled for appointment type and age in the model, yet women were still more likely to reveal a CSI to *some* undergraduates than men. This pattern holds for CSIs in aggregate as well as specifically for depression, first-generation status, and growing up in a low-income household, while trending in the same direction for other identities. While perceived level of stigmatization has been shown to encourage concealment of CSIs in general [23,91,92], our data suggest the opposite: that increased perceived stigmatization may be encouraging women to emerge as role models.

We posit that women are more open about their CSIs in part because they have encountered bias in S&E [93–96] and therefore may recognize the importance of instructor role models for students. Specifically, women face gender bias across all levels of academia including during hiring [97,98], tenure and promotion decisions [99], compensation [100], and in negative attitudes and “snide comments” [101]. Further, women are more likely to have their work devalued by both men and women [102] and are credited less via authorship [103]. However, at the undergraduate level, women in S&E courses report increased course success and persistence in science majors from having a same-gender instructor [104,105] or role model of a successful scientist [106,107]. Therefore, women’s own experiences with bias and the impact that a role model may have had on their own trajectories might explain their willingness to reveal CSIs to provide undergraduates with a same- or similar-identity role model. Prior experiences of bias in S&E based on gender likely also explain why women rated each of the CSIs as more stigmatized than men; previous exposure to bias can make people more perceptive to it in other situations [108,109].

Women disclosing CSIs more than men may also be due to students’ expectations for warmer relationships with women instructors. Students and mentees expect more personable and stronger psychosocial relationships with women than they do men [44,45]. Potentially due to the influence of students’ evaluations of teaching on promotion and tenure decision [110–112], women may strive to meet these expectations. Sharing personal details, including CSIs, may be a way to help foster a strong connection [16]. However, if women are choosing to reveal CSIs to their undergraduates to meet these gendered expectations, it is adding unpaid, emotional labor that their male colleagues without these gendered expectations do not have to undertake. Therefore, some women may reveal an identity because they prefer to live authentically or be open about their CSI while others may reveal because students will have a higher regard for the instructor if she seems more personable [23,33]. The impact on the instructor of revealing a CSI may depend, at least in part, on her underlying motivation to reveal the identity. Revealing a CSI may be perceived more negatively if the instructor reveals an identity to meet students’ expectations in cases where she seems uncomfortable sharing the identity [113].

Women were specifically more likely to reveal CSIs to *some* students than men, but we found no gender differences in revealing CSIs to *all* students. Instances where an instructor reveals a CSI to some students is likely occurring during office hours or in one-on-one discussions before or after class. Undergraduates request and expect to be granted special favors, such as extra credit and accepting late work, from women more than men [114]; these requests likely come during office hours or before and after class and may drive higher office hour attendance for women instructors. Additionally, for the same amount of student-instructor interaction, students rate women lower on interpersonal measures than men [115], which may be because students expect a greater level of personal support from women instructors [44,45]. This expectation for a more personal relationship with women may lead students to share more about themselves, including CSIs, with women, potentially leading to women revealing their own CSIs to students in these one-on-one or small group settings outside of class. Therefore, women being more likely to reveal a CSI to some students than men may be due to differences in the frequency of interactions with students, student motivations for seeking support outside of class, or even students sharing more about their identities with women rather than men. Future research could explore these interactions in office hours in more depth to better understand the impetus and context of these situations where instructors reveal their identities.

Of note, men are both more likely to be first-generation college students and less likely to disclose it. This illustrates a specific instance where women are driving the representation of first-generation college students among S&E instructors despite being less likely to report this identity. Despite any potential consequences, this pattern highlights the deliberate effort from women to highlight CSIs to S&E undergraduates.

While individuals with CSIs are generally less likely to reveal an identity if they anticipate greater stigma [38,39], women instructors in S&E demonstrate the inverse: women perceive greater stigma and reveal CSIs to undergraduates more than men. This is true overall as well as specifically for revealing depression, first-generation status, and growing up in a low-income household. This unexpected pattern may be due to the powerful impact that women role models in science can have on other women and girls [106,107,116,117] and these instructors intend to provide that role model for students based on other identities regardless of any subsequent stigma they encounter. However, as stigmatized identities carry negative stereotypes, if there are negative consequences from revealing these identities, then women may disproportionately face those consequences in addition to the gender biases already prevalent in S&E [93,94,96].

### Limitations and future directions

In this study, we focus on differences between men and women and do not discuss any patterns among responses from non-binary individuals. Excluding non-binary individuals from our analyses removes a participant population that is vastly understudied and not well-understood in professional S&E contexts [80,118], but we chose to focus on the experiences of men and women as the majority of the participants fell into the gender binary and doing so allowed us to focus on gender differences between men and women exclusively and exclude any influence of homophobia or bias against non-binary or genderqueer individuals.

Throughout our analyses, we did not assess the impact of multiple identities (i.e., a racial identity and a gender identity) on whether an instructor revealed a CSI or their perceived stigma. The intersectional systems of oppression that women of color experience result in different experiences in S&E than those of white women [13,119,120]. However, although we did not have the statistical power to explore these interactions (i.e., run a model with gender*race/ethnicity as an interactive term), we did control for race/ethnicity as well as age and appointment in our models which allows us to make inferences based on the gender differences described. Additionally, because the majority (72%) of participants were white, the results described may not be generalizable to a more racially and ethnically diverse sample. However, the uneven distribution of racial/ethnic identities is reflective of the disparities that exist within S&E faculty [121,122]. Future work can build on these results and explore these interactions along gender and racial/ethnic identities as well as use a sampling design to specifically recruit a more racially and ethnically diverse sample of instructors. In our analyses we did not account for academic institution or state, as the distribution of the data across institutions and states was not adequate to assess such differences. The participants spanned 46 states, so although there are likely regional differences in revealing CSIs and perceived stigma, we posit that the results presented in the present study are generally reflective of the U.S.

We took steps to ensure a random sample participated in our study by recruiting instructors from each very high research activity doctoral granting institution across science and engineering departments in the U.S. Instructors did not know that we were going to be asking about any of these identities to limit sampling bias towards any of these identities. However, there may be some degree of sampling bias because faculty often receive many requests per day and may not have interest in completing a survey. Additionally, the recruitment email included the language “help improve STEM education” and that participating in the survey would help gain insight into who the faculty and instructors who teach undergraduates in science and engineering are in order to better understand potential gaps between those teaching and those learning undergraduate science and engineering. This language may have created sampling bias and led to more instructors invested in diversity, equity, and inclusion initiatives or who practice evidence-based teaching participating than their colleagues. We worked to counteract this bias and increase participation by providing incentives and reminders to individuals who did not complete the survey after the first recruitment, but we may be overestimating the percentage of faculty who reveal these identities if they were more interested in participating in this survey.

Future studies can explore the extent to which gendered experiences or expectations consciously inform women’s decisions to reveal CSIs to students and to what extent perceived stigma associated with their identity or in S&E generally influences their decisions.

## Conclusion

In conclusion, we find that women reveal CSIs more readily than men despite also perceiving greater stigma associated with these identities. Specifically, women reveal that they have depression, are a first-generation college student, and grew up in a low-income household to some undergraduates more than men. While this indicates that women are positioning themselves to be role models for students based on a variety of identities, our findings encourage efforts to identify barriers instructors face to revealing CSIs to their students and understand the potential impact that revealing these identities has on undergraduate science and engineering students.

## Supporting information

S1 Fig Percent of men and women who reveal each of the identities to all, some, or none of their undergraduate students. (TIF)

S1 Table Demographic breakdown of participants. (DOCX)

S2 Table Results from logistic regressions for reporting each of the CSIs. Group of interest is in parentheses and reference groups are men, white, <50, and lecturers. Odds ratio (OR) calculated by exponentiating the beta.
(DOCX)

S3 Table Results from multinomial regressions predicting revealing CSIs overall and each of the CSIs specifically (reference group: Revealing to no undergraduates). Group of interest is in parentheses and reference groups are men, white, <50, and lecturers. Odds ratio (OR) calculated by exponentiating the beta.
(DOCX)

S4 Table Results from ordinal regressions predicting stigma ratings for CSIs overall and each of the CSIs specifically. Group of interest is in parentheses and reference groups are men, white, <50, and lecturers. Odds ratio (OR) calculated by exponentiating the beta.
(DOCX)

S1 Appendix Copy of survey questions analyzed. (DOCX)

S2 Appendix De-identified data. (CSV)
