# Supplementary figures and images for: Women drive efforts to highlight concealable stigmatized identities in U.S. academic science and engineering
Source: PLoS One. 2023 Jul 19;18(7):e0287795. doi: 10.1371/journal.pone.0287795 (PMC10355415; doi:10.1371/journal.pone.0287795)

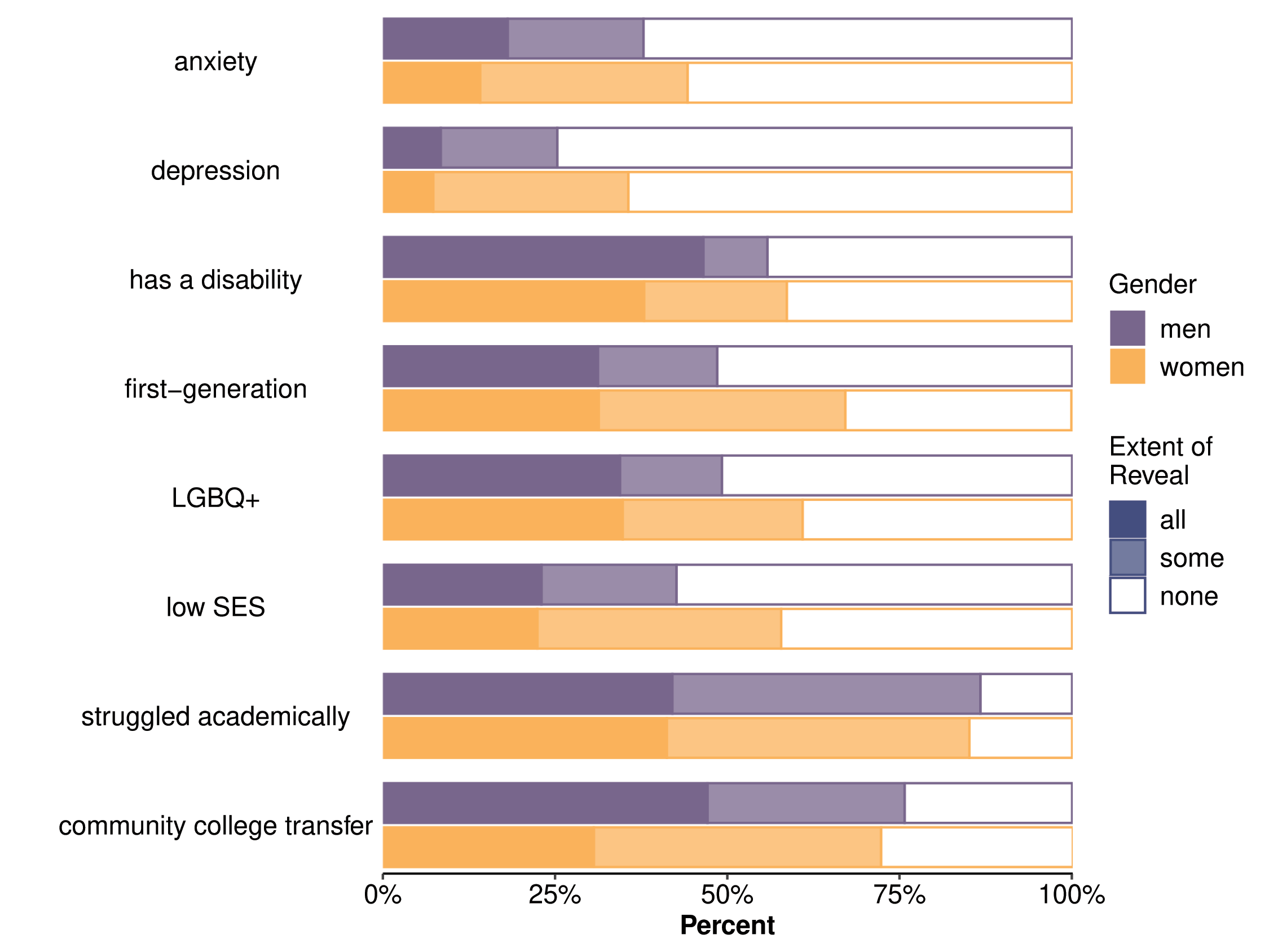

Supplement: S1 Fig — (TIF) [file pone.0287795.s001.tif]
