# Supplementary material for: Women drive efforts to highlight concealable stigmatized identities in U.S. academic science and engineering
Source: PLoS One. 2023 Jul 19;18(7):e0287795. doi: 10.1371/journal.pone.0287795 (PMC10355415; doi:10.1371/journal.pone.0287795)
Supplement: S1 Table — (DOCX) [file pone.0287795.s002.docx]

**S1 Table.** Demographic breakdown of participants.

*Note: In order to preserve the anonymity of participants, we do not provide disaggregated demographic information for gender-queer or non-binary individuals.*

| **Identity** | **Group** | **Demographic** | **Percent (n)** |
| --- | --- | --- | --- |
| **LGBQ+** | Overall | No | 94.65 (1821) |
|  |  | Yes | 5.35 (103) |
|  | Men | No | 95.43 (1106) |
|  |  | Yes | 4.57 (53) |
|  | Women | No | 94.54 (710) |
|  |  | Yes | 5.46 (41) |
| **Race/ ethnicity** | Overall | White | 72.08 (1451) |
|  |  | Asian | 13.02 (262) |
|  |  | Hispanic, Latino/A, Or Of Spanish Origin | 4.47 (90) |
|  |  | Other (Including Multiracial), Please Describe | 2.83 (57) |
|  |  | Decline To State | 5.61 (113) |
|  |  | Black Or African American | 1.59 (32) |
|  |  | American Indian Or Alaska Native | 0.25 (5) |
|  |  | Pacific Islander | 0.1 (2) |
|  |  | Native Hawaiian | 0.05 (1) |
|  | Men | American Indian Or Alaska Native | 0.26 (3) |
|  |  | Asian | 15.19 (178) |
|  |  | Black Or African American | 1.02 (12) |
|  |  | Decline To State | 3.07 (36) |
|  |  | Hispanic, Latino/A, Or Of Spanish Origin | 4.44 (52) |
|  |  | Native Hawaiian | 0 (0) |
|  |  | Other (Including Multiracial), Please Describe | 2.9 (34) |
|  |  | Pacific Islander | 0.09 (1) |
|  |  | White | 73.04 (856) |
|  | Women | American Indian Or Alaska Native | 0.26 (2) |
|  |  | Asian | 10.79 (83) |
|  |  | Black Or African American | 2.6 (20) |
|  |  | Decline To State | 2.99 (23) |
|  |  | Hispanic, Latino/A, Or Of Spanish Origin | 4.81 (37) |
|  |  | Native Hawaiian | 0.13 (1) |
|  |  | Other (Including Multiracial), Please Describe | 2.73 (21) |
|  |  | Pacific Islander | 0.13 (1) |
|  |  | White | 75.55 (581) |
| **Depression** | Overall | Someone Who Does Not Have Depression | 67.21 (1353) |
|  |  | Someone Who Has Or Has Had Depression | 23.94 (482) |
|  |  | Decline To State | 8.84 (178) |
|  | Men | Decline To State | 6.57 (77) |
|  |  | Someone Who Does Not Have Depression | 73.21 (858) |
|  |  | Someone Who Has Or Has Had Depression | 20.22 (237) |
|  | Women | Decline To State | 6.50 (50) |
|  |  | Someone Who Does Not Have Depression | 63.07 (485) |
|  |  | Someone Who Has Or Has Had Depression | 30.43 (234) |
| **Anxiety** | Overall | Someone Who Does Not Have Anxiety | 59.41 (1196) |
|  |  | Someone Who Has Or Has Had Anxiety | 32.84 (661) |
|  |  | Decline To State | 7.75 (156) |
|  | Men | Decline To State | 5.63 (66) |
|  |  | Someone Who Does Not Have Anxiety | 67.06 (786) |
|  |  | Someone Who Has Or Has Had Anxiety | 27.3 (320) |
|  | Women | Decline To State | 5.46 (42) |
|  |  | Someone Who Does Not Have Anxiety | 52.02 (400) |
|  |  | Someone Who Has Or Has Had Anxiety | 42.52 (327) |
| **Socio-economic status** | Overall | Middle income | 66.22 (1333) |
|  |  | Low income | 19.13 (385) |
|  |  | Upper income | 8.99 (181) |
|  |  | Decline To State | 5.66 (114) |
|  | Men | Decline To State | 3.41 (40) |
|  |  | Low income | 19.71 (231) |
|  |  | Middle income | 68 (797) |
|  |  | Upper income | 8.87 (104) |
|  | Women | Decline To State | 2.99 (23) |
|  |  | Low income | 19.12 (147) |
|  |  | Middle income | 68.14 (524) |
|  |  | Upper income | 9.75 (75) |
| **College generation status** | Overall | A Non-First-Generation College Student (At Least One Of My Parents Or Guardians Has A Bachelor‚Äôs Degree Or Equivalent) | 66.87 (1346) |
|  |  | A First-Generation College Student | 28.81 (580) |
|  |  | Decline To State | 4.32 (87) |
|  | Men | A First-Generation College Student | 31.57 (370) |
|  |  | A Non-First-Generation College Student (At Least One Of My Parents Or Guardians Has A Bachelor‚Äôs Degree Or Equivalent) | 66.3 (777) |
|  |  | Decline To State | 2.13 (25) |
|  | Women | A First-Generation College Student | 26.27 (202) |
|  |  | A Non-First-Generation College Student (At Least One Of My Parents Or Guardians Has A Bachelor’s Degree Or Equivalent) | 71.65 (551) |
|  |  | Decline To State | 2.08 (16) |
| **Academic struggle in college** | Overall | Someone Who Did Not Struggle Academically In College | 82.02 (1651) |
|  |  | Someone Who Struggled Academically In College | 13.26 (267) |
|  |  | Decline To State | 4.72 (95) |
|  | Men | Decline To State | 2.64 (31) |
|  |  | Someone Who Did Not Struggle Academically In College | 84.56 (991) |
|  |  | Someone Who Struggled Academically In College | 12.8 (150) |
|  | Women | Decline To State | 2.73 (21) |
|  |  | Someone Who Did Not Struggle Academically In College | 82.44 (634) |
|  |  | Someone Who Struggled Academically In College | 14.82 (114) |
| **Disability status** | Overall | Not Having A Disability | 88.87 (1789) |
|  |  | Having A Disability | 5.37 (108) |
|  |  | Decline To State | 5.76 (116) |
|  | Men | Decline To State | 3.41 (40) |
|  |  | Having A Disability | 3.75 (44) |
|  |  | Not Having A Disability | 92.83 (1088) |
|  | Women | Decline To State | 3.77 (29) |
|  |  | Having A Disability | 7.67 (59) |
|  |  | Not Having A Disability | 88.56 (681) |
| **Community college transfer status** | Overall | I Started My College Career At The Same 4-Year Institution From Which I Graduated | 80.23 (1615) |
|  |  | I Transferred To A 4-Year Institution From Another 4-Year Institution | 6.81 (137) |
|  |  | I Transferred To A 4-Year Institution From A 2-Year College, A Community College, A Junior College, Or A Technical College | 5.41 (109) |
|  |  | If None Of The Above Reflect Your Experience, Please Describe | 3.63 (73) |
|  |  | Decline To State | 3.92 (79) |
|  | Men | Decline To State | 2.13 (25) |
|  |  | I Started My College Career At The Same 4-Year Institution From Which I Graduated | 81.66 (957) |
|  |  | I Transferred To A 4-Year Institution From A 2-Year College, A Community College, A Junior College, Or A Technical College | 6.06 (71) |
|  |  | I Transferred To A 4-Year Institution From Another 4-Year Institution | 6.31 (74) |
|  |  | If None Of The Above Reflect Your Experience, Please Describe | 3.84 (45) |
|  | Women | Decline To State | 1.95 (15) |
|  |  | I Started My College Career At The Same 4-Year Institution From Which I Graduated | 81.79 (629) |
|  |  | I Transferred To A 4-Year Institution From A 2-Year College, A Community College, A Junior College, Or A Technical College | 4.68 (36) |
|  |  | I Transferred To A 4-Year Institution From Another 4-Year Institution | 7.93 (61) |
|  |  | If None Of The Above Reflect Your Experience, Please Describe | 3.64 (28) |
