# Supplementary material for: Women drive efforts to highlight concealable stigmatized identities in U.S. academic science and engineering
Source: PLoS One. 2023 Jul 19;18(7):e0287795. doi: 10.1371/journal.pone.0287795 (PMC10355415; doi:10.1371/journal.pone.0287795)
Supplement: S2 Table — Group of interest is in parentheses and reference groups are men, white, <50, and lecturers. Odds ratio (OR) calculated by exponentiating the beta. (DOCX) [file pone.0287795.s003.docx]

**S2 Table.** Results from logistic regressions for reporting each of the CSIs. Group of interest is in parentheses and reference groups are men, white, <50, and lecturers. Odds ratio (OR) calculated by exponentiating the beta.

| **CSI** | **Predictor** | **Beta** | **SE** | ***pval*** | **Std Beta** | **OR** |
| --- | --- | --- | --- | --- | --- | --- |
| **LGBQ+** | (Intercept) | 0.066 | 0.013 | 0.000 | 0.051 | 1.068 |
|  | Gender (woman) | 0.004 | 0.011 | 0.755 | 0.002 | 1.004 |
|  | Race (Asian) | -0.050 | 0.016 | 0.002 | -0.017 | 0.951 |
|  | Race (PEER) | -0.007 | 0.021 | 0.734 | -0.002 | 0.993 |
|  | Age (50+) | -0.032 | 0.012 | 0.009 | -0.016 | 0.969 |
|  | Appointment (tenured) | 0.000 | 0.013 | 0.992 | 0.000 | 1.000 |
|  | Appointment (tenure-track) | 0.023 | 0.016 | 0.153 | 0.009 | 1.023 |
| **Depression** | (Intercept) | 0.303 | 0.026 | 0.000 | 0.263 | 1.354 |
|  | Gender (woman) | 0.076 | 0.022 | 0.001 | 0.037 | 1.079 |
|  | Race (Asian) | -0.157 | 0.032 | 0.000 | -0.053 | 0.855 |
|  | Race (PEER) | 0.007 | 0.042 | 0.865 | 0.002 | 1.007 |
|  | Age (50+) | -0.066 | 0.024 | 0.007 | -0.033 | 0.936 |
|  | Appointment (tenured) | -0.051 | 0.026 | 0.044 | -0.026 | 0.950 |
|  | Appointment (tenure-track) | 0.018 | 0.032 | 0.570 | 0.007 | 1.019 |
| **Anxiety** | (Intercept) | 0.419 | 0.027 | 0.000 | 0.356 | 1.520 |
|  | Gender (woman) | 0.116 | 0.024 | 0.000 | 0.057 | 1.123 |
|  | Race (Asian) | -0.157 | 0.034 | 0.000 | -0.053 | 0.855 |
|  | Race (PEER) | -0.028 | 0.045 | 0.537 | -0.007 | 0.973 |
|  | Age (50+) | -0.147 | 0.026 | 0.000 | -0.073 | 0.863 |
|  | Appointment (tenured) | -0.065 | 0.027 | 0.017 | -0.033 | 0.937 |
|  | Appointment (tenure-track) | 0.035 | 0.034 | 0.306 | 0.014 | 1.036 |
| **Low SES** | (Intercept) | 0.163 | 0.023 | 0.000 | 0.198 | 1.177 |
|  | Gender (woman) | 0.003 | 0.020 | 0.867 | 0.002 | 1.003 |
|  | Race (Asian) | 0.037 | 0.029 | 0.201 | 0.013 | 1.038 |
|  | Race (PEER) | 0.097 | 0.038 | 0.011 | 0.025 | 1.101 |
|  | Age (50+) | 0.021 | 0.022 | 0.342 | 0.010 | 1.021 |
|  | Appointment (tenured) | 0.015 | 0.023 | 0.525 | 0.007 | 1.015 |
|  | Appointment (tenure-track) | 0.028 | 0.029 | 0.345 | 0.011 | 1.028 |
| **First-gen** | (Intercept) | 0.285 | 0.027 | 0.000 | 0.299 | 1.330 |
|  | Gender (woman) | -0.050 | 0.023 | 0.031 | -0.025 | 0.951 |
|  | Race (Asian) | 0.073 | 0.033 | 0.028 | 0.025 | 1.075 |
|  | Race (PEER) | 0.090 | 0.043 | 0.039 | 0.023 | 1.094 |
|  | Age (50+) | 0.074 | 0.025 | 0.003 | 0.037 | 1.077 |
|  | Appointment (tenured) | -0.032 | 0.026 | 0.223 | -0.016 | 0.968 |
|  | Appointment (tenure-track) | 0.015 | 0.033 | 0.653 | 0.006 | 1.015 |
| **Academic struggle** | (Intercept) | 0.193 | 0.020 | 0.000 | 0.135 | 1.212 |
|  | Gender (woman) | 0.005 | 0.017 | 0.794 | 0.002 | 1.005 |
|  | Race (Asian) | -0.070 | 0.025 | 0.005 | -0.024 | 0.933 |
|  | Race (PEER) | 0.094 | 0.033 | 0.004 | 0.024 | 1.099 |
|  | Age (50+) | 0.005 | 0.019 | 0.771 | 0.003 | 1.005 |
|  | Appointment (tenured) | -0.092 | 0.020 | 0.000 | -0.046 | 0.912 |
|  | Appointment (tenure-track) | -0.061 | 0.025 | 0.013 | -0.025 | 0.940 |
| **Disability** | (Intercept) | 0.037 | 0.013 | 0.005 | 0.055 | 1.038 |
|  | Gender (woman) | 0.043 | 0.012 | 0.000 | 0.021 | 1.044 |
|  | Race (Asian) | -0.034 | 0.016 | 0.037 | -0.012 | 0.967 |
|  | Race (PEER) | 0.038 | 0.022 | 0.079 | 0.010 | 1.039 |
|  | Age (50+) | -0.001 | 0.013 | 0.922 | -0.001 | 0.999 |
|  | Appointment (tenured) | -0.007 | 0.013 | 0.579 | -0.004 | 0.993 |
|  | Appointment (tenure-track) | 0.033 | 0.017 | 0.047 | 0.013 | 1.033 |
| **CC transfer** | (Intercept) | 0.090 | 0.013 | 0.000 | 0.055 | 1.094 |
|  | Gender (woman) | -0.023 | 0.012 | 0.044 | -0.011 | 0.977 |
|  | Race (Asian) | -0.019 | 0.017 | 0.249 | -0.006 | 0.981 |
|  | Race (PEER) | 0.025 | 0.022 | 0.243 | 0.006 | 1.026 |
|  | Age (50+) | -0.007 | 0.013 | 0.601 | -0.003 | 0.993 |
|  | Appointment (tenured) | -0.036 | 0.013 | 0.007 | -0.018 | 0.965 |
|  | Appointment (tenure-track) | -0.022 | 0.017 | 0.197 | -0.009 | 0.979 |
