# Supplementary material for: Women drive efforts to highlight concealable stigmatized identities in U.S. academic science and engineering
Source: PLoS One. 2023 Jul 19;18(7):e0287795. doi: 10.1371/journal.pone.0287795 (PMC10355415; doi:10.1371/journal.pone.0287795)
Supplement: S3 Table — Group of interest is in parentheses and reference groups are men, white, <50, and lecturers. Odds ratio (OR) calculated by exponentiating the beta. (DOCX) [file pone.0287795.s004.docx]

**S3 Table.** Results from multinomial regressions predicting revealing CSIs overall and each of the CSIs specifically (reference group: revealing to no undergraduates). Group of interest is in parentheses and reference groups are men, white, <50, and lecturers. Odds ratio (OR) calculated by exponentiating the beta.

| **CSI** | **Extent of reveal** | **Predictor** | **Beta** | ***pval*** | **SE** | **OR** |
| --- | --- | --- | --- | --- | --- | --- |
| **Aggregate** | some | Gender (woman) | 0.38 | 0.00 | 0.10 | 1.46 |
|  |  | Race (Asian) | -0.85 | 0.00 | 0.20 | 0.43 |
|  |  | Race (PEER) | -0.35 | 0.06 | 0.18 | 0.70 |
|  |  | Age (50+) | -0.22 | 0.07 | 0.12 | 0.81 |
|  |  | Appointment (tenured) | -0.91 | 0.00 | 0.12 | 0.40 |
|  |  | Appointment (tenure-track) | -1.07 | 0.00 | 0.14 | 0.34 |
|  | all | Gender (woman) | -0.04 | 0.77 | 0.13 | 0.96 |
|  |  | Race (Asian) | -1.15 | 0.00 | 0.28 | 0.32 |
|  |  | Race (PEER) | -0.17 | 0.44 | 0.22 | 0.84 |
|  |  | Age (50+) | -0.10 | 0.49 | 0.14 | 0.91 |
|  |  | Appointment (tenured) | -0.60 | 0.00 | 0.15 | 0.55 |
|  |  | Appointment (tenure-track) | -0.75 | 0.00 | 0.18 | 0.47 |
| **LGBQ+** | some | Gender (woman) | 1.16 | 0.06 | 0.62 | 3.20 |
|  |  | Race (Asian) | -33.88 | 0.00 | 0.00 | 0.00 |
|  |  | Race (PEER) | 1.53 | 0.12 | 0.99 | 4.64 |
|  |  | Age (50+) | 20.46 | 0.00 | 0.39 | 7.71E+08 |
|  |  | Appointment (tenured) | -21.27 | 0.00 | 0.39 | 0.00 |
|  |  | Appointment (tenure-track) | 0.86 | 0.24 | 0.74 | 2.37 |
|  | all | Gender (woman) | 0.58 | 0.31 | 0.57 | 1.78 |
|  |  | Race (Asian) | -24.14 | 0.00 | 0.00 | 0.00 |
|  |  | Race (PEER) | 0.13 | 0.92 | 1.30 | 1.14 |
|  |  | Age (50+) | -0.35 | 0.69 | 0.87 | 0.70 |
|  |  | Appointment (tenured) | -0.55 | 0.48 | 0.78 | 0.58 |
|  |  | Appointment (tenure-track) | 0.65 | 0.38 | 0.75 | 1.92 |
| **Depression** | some | Gender (woman) | 0.66 | 0.01 | 0.25 | 1.93 |
|  |  | Race (Asian) | -0.78 | 0.18 | 0.58 | 0.46 |
|  |  | Race (PEER) | -1.48 | 0.02 | 0.63 | 0.23 |
|  |  | Age (50+) | -0.27 | 0.33 | 0.28 | 0.76 |
|  |  | Appointment (tenured) | -1.06 | 0.00 | 0.28 | 0.35 |
|  |  | Appointment (tenure-track) | -1.57 | 0.00 | 0.36 | 0.21 |
|  | all | Gender (woman) | 0.16 | 0.72 | 0.44 | 1.17 |
|  |  | Race (Asian) | -0.59 | 0.58 | 1.05 | 0.56 |
|  |  | Race (PEER) | -0.19 | 0.81 | 0.77 | 0.83 |
|  |  | Age (50+) | -0.54 | 0.28 | 0.50 | 0.58 |
|  |  | Appointment (tenured) | 0.03 | 0.95 | 0.50 | 1.03 |
|  |  | Appointment (tenure-track) | -1.13 | 0.11 | 0.70 | 0.32 |
| **Anxiety** | some | Gender (woman) | 0.29 | 0.17 | 0.21 | 1.33 |
|  |  | Race (Asian) | -1.59 | 0.00 | 0.54 | 0.20 |
|  |  | Race (PEER) | -1.01 | 0.03 | 0.47 | 0.36 |
|  |  | Age (50+) | -0.56 | 0.02 | 0.25 | 0.57 |
|  |  | Appointment (tenured) | -0.89 | 0.00 | 0.24 | 0.41 |
|  |  | Appointment (tenure-track) | -1.12 | 0.00 | 0.28 | 0.33 |
|  | all | Gender (woman) | -0.25 | 0.45 | 0.33 | 0.78 |
|  |  | Race (Asian) | -0.75 | 0.24 | 0.63 | 0.47 |
|  |  | Race (PEER) | -0.40 | 0.53 | 0.64 | 0.67 |
|  |  | Age (50+) | -1.25 | 0.01 | 0.45 | 0.29 |
|  |  | Appointment (tenured) | -0.60 | 0.11 | 0.37 | 0.55 |
|  |  | Appointment (tenure-track) | -1.22 | 0.01 | 0.45 | 0.30 |
| **Low SES** | some | Gender (woman) | 0.69 | 0.02 | 0.29 | 1.99 |
|  |  | Race (Asian) | -0.85 | 0.09 | 0.49 | 0.43 |
|  |  | Race (PEER) | -0.52 | 0.32 | 0.52 | 0.60 |
|  |  | Age (50+) | -0.20 | 0.51 | 0.31 | 0.82 |
|  |  | Appointment (tenured) | -0.63 | 0.06 | 0.33 | 0.53 |
|  |  | Appointment (tenure-track) | -1.54 | 0.00 | 0.47 | 0.21 |
|  | all | Gender (woman) | 0.13 | 0.72 | 0.36 | 1.14 |
|  |  | Race (Asian) | -0.98 | 0.09 | 0.59 | 0.38 |
|  |  | Race (PEER) | -0.12 | 0.83 | 0.56 | 0.89 |
|  |  | Age (50+) | 0.13 | 0.75 | 0.40 | 1.14 |
|  |  | Appointment (tenured) | -0.32 | 0.47 | 0.45 | 0.72 |
|  |  | Appointment (tenure-track) | 0.20 | 0.69 | 0.50 | 1.22 |
| **First-gen** | some | Gender (woman) | 0.81 | 0.00 | 0.25 | 2.25 |
|  |  | Race (Asian) | -0.84 | 0.02 | 0.36 | 0.43 |
|  |  | Race (PEER) | -0.17 | 0.70 | 0.45 | 0.84 |
|  |  | Age (50+) | -0.72 | 0.01 | 0.27 | 0.49 |
|  |  | Appointment (tenured) | -0.81 | 0.00 | 0.28 | 0.44 |
|  |  | Appointment (tenure-track) | -0.95 | 0.01 | 0.34 | 0.39 |
|  | all | Gender (woman) | 0.49 | 0.07 | 0.27 | 1.63 |
|  |  | Race (Asian) | -1.73 | 0.00 | 0.55 | 0.18 |
|  |  | Race (PEER) | 0.28 | 0.51 | 0.43 | 1.33 |
|  |  | Age (50+) | -0.59 | 0.04 | 0.29 | 0.55 |
|  |  | Appointment (tenured) | -0.70 | 0.02 | 0.31 | 0.50 |
|  |  | Appointment (tenure-track) | -1.05 | 0.01 | 0.39 | 0.35 |
| **Academic struggle** | some | Gender (woman) | -0.69 | 0.12 | 0.45 | 0.50 |
|  |  | Race (Asian) | -0.54 | 0.50 | 0.79 | 0.58 |
|  |  | Race (PEER) | 0.41 | 0.50 | 0.61 | 1.50 |
|  |  | Age (50+) | -0.62 | 0.23 | 0.51 | 0.54 |
|  |  | Appointment (tenured) | -1.09 | 0.04 | 0.53 | 0.34 |
|  |  | Appointment (tenure-track) | -1.69 | 0.01 | 0.61 | 0.18 |
|  | all | Gender (woman) | -0.52 | 0.27 | 0.47 | 0.59 |
|  |  | Race (Asian) | -0.76 | 0.39 | 0.88 | 0.47 |
|  |  | Race (PEER) | -0.49 | 0.49 | 0.71 | 0.62 |
|  |  | Age (50+) | -0.09 | 0.87 | 0.53 | 0.92 |
|  |  | Appointment (tenured) | -1.01 | 0.07 | 0.56 | 0.36 |
|  |  | Appointment (tenure-track) | -1.44 | 0.03 | 0.66 | 0.24 |
| **Disability** | some | Gender (woman) | 0.64 | 0.43 | 0.80 | 1.89 |
|  |  | Race (Asian) | -18.22 | 0.00 | 0.00 | 0.00 |
|  |  | Race (PEER) | -1.10 | 0.34 | 1.16 | 0.33 |
|  |  | Age (50+) | 1.49 | 0.14 | 1.00 | 4.45 |
|  |  | Appointment (tenured) | -1.92 | 0.06 | 1.01 | 0.15 |
|  |  | Appointment (tenure-track) | -0.53 | 0.53 | 0.85 | 0.59 |
|  | all | Gender (woman) | -0.65 | 0.32 | 0.65 | 0.52 |
|  |  | Race (Asian) | -19.63 | 0.00 | 0.00 | 0.00 |
|  |  | Race (PEER) | -16.72 | 0.00 | 0.00 | 0.00 |
|  |  | Age (50+) | 1.16 | 0.17 | 0.84 | 3.20 |
|  |  | Appointment (tenured) | -1.29 | 0.13 | 0.84 | 0.28 |
|  |  | Appointment (tenure-track) | -1.11 | 0.19 | 0.83 | 0.33 |
| **CC transfer** | some | Gender (woman) | -0.28 | 0.66 | 0.64 | 0.75 |
|  |  | Race (Asian) | -1.55 | 0.14 | 1.06 | 0.21 |
|  |  | Race (PEER) | -1.61 | 0.21 | 1.29 | 0.20 |
|  |  | Age (50+) | 0.46 | 0.53 | 0.73 | 1.58 |
|  |  | Appointment (tenured) | -2.04 | 0.01 | 0.78 | 0.13 |
|  |  | Appointment (tenure-track) | -0.45 | 0.55 | 0.75 | 0.64 |
|  | all | Gender (woman) | -1.28 | 0.12 | 0.82 | 0.28 |
|  |  | Race (Asian) | -16.20 | 0.00 | 0.00 | 0.00 |
|  |  | Race (PEER) | -1.22 | 0.33 | 1.26 | 0.29 |
|  |  | Age (50+) | 0.59 | 0.47 | 0.81 | 1.81 |
|  |  | Appointment (tenured) | -1.11 | 0.19 | 0.85 | 0.33 |
|  |  | Appointment (tenure-track) | -0.23 | 0.82 | 0.98 | 0.79 |
