# Supplementary material for: Women drive efforts to highlight concealable stigmatized identities in U.S. academic science and engineering
Source: PLoS One. 2023 Jul 19;18(7):e0287795. doi: 10.1371/journal.pone.0287795 (PMC10355415; doi:10.1371/journal.pone.0287795)
Supplement: S4 Table — Group of interest is in parentheses and reference groups are men, white, <50, and lecturers. Odds ratio (OR) calculated by exponentiating the beta. (DOCX) [file pone.0287795.s005.docx]

**S4 Table.** Results from ordinal regressions predicting stigma ratings for CSIs overall and each of the CSIs specifically. Group of interest is in parentheses and reference groups are men, white, <50, and lecturers. Odds ratio (OR) calculated by exponentiating the beta.

| **CSI** | **Predictor** | **Beta** | **SE** | ***zval*** | ***pval*** | **OR** |
| --- | --- | --- | --- | --- | --- | --- |
| **Aggregate** | Gender (woman) | 0.657 | 0.034 | 13.603 | 0.000 | 1.929 |
|  | Race (Asian) | -0.558 | 0.050 | -11.068 | 0.000 | 0.572 |
|  | Race (PEER) | 0.517 | 0.064 | 8.125 | 0.000 | 1.677 |
|  | Age (50+) | -0.407 | 0.037 | -11.147 | 0.000 | 0.666 |
|  | Appointment (tenured) | -0.129 | 0.038 | -3.414 | 0.001 | 0.879 |
|  | Appointment (tenure-track) | -0.005 | 0.048 | -0.107 | 0.915 | 0.995 |
| **LGBQ+** | Gender (woman) | 0.670 | 0.092 | 7.245 | 0.000 | 1.955 |
|  | Race (Asian) | -0.220 | 0.139 | -1.582 | 0.114 | 0.802 |
|  | Race (PEER) | 0.574 | 0.175 | 3.277 | 0.001 | 1.775 |
|  | Age (50+) | -0.069 | 0.101 | -0.686 | 0.493 | 0.933 |
|  | Appointment (tenured) | -0.051 | 0.105 | -0.489 | 0.625 | 0.950 |
|  | Appointment (tenure-track) | 0.018 | 0.131 | 0.136 | 0.892 | 1.018 |
| **Depression** | Gender (woman) | 0.526 | 0.094 | 5.595 | 0.000 | 1.693 |
|  | Race (Asian) | -0.708 | 0.140 | -5.057 | 0.000 | 0.492 |
|  | Race (PEER) | 0.658 | 0.183 | 3.590 | 0.000 | 1.931 |
|  | Age (50+) | -0.465 | 0.102 | -4.543 | 0.000 | 0.628 |
|  | Appointment (tenured) | 0.051 | 0.106 | 0.482 | 0.630 | 1.053 |
|  | Appointment (tenure-track) | -0.016 | 0.135 | -0.122 | 0.903 | 0.984 |
| **Anxiety** | Gender (woman) | 0.592 | 0.095 | 6.245 | 0.000 | 1.808 |
|  | Race (Asian) | -0.517 | 0.141 | -3.666 | 0.000 | 0.596 |
|  | Race (PEER) | 0.689 | 0.184 | 3.742 | 0.000 | 1.992 |
|  | Age (50+) | -0.315 | 0.103 | -3.066 | 0.002 | 0.730 |
|  | Appointment (tenured) | -0.063 | 0.107 | -0.588 | 0.557 | 0.939 |
|  | Appointment (tenure-track) | 0.013 | 0.135 | 0.098 | 0.922 | 1.013 |
| **Low SES** | Gender (woman) | 0.816 | 0.100 | 8.128 | 0.000 | 2.262 |
|  | Race (Asian) | -0.720 | 0.163 | -4.423 | 0.000 | 0.487 |
|  | Race (PEER) | 0.631 | 0.183 | 3.457 | 0.001 | 1.879 |
|  | Age (50+) | -0.698 | 0.112 | -6.245 | 0.000 | 0.498 |
|  | Appointment (tenured) | -0.179 | 0.115 | -1.553 | 0.120 | 0.836 |
|  | Appointment (tenure-track) | -0.031 | 0.141 | -0.221 | 0.825 | 0.969 |
| **First-gen** | Gender (woman) | 0.867 | 0.110 | 7.898 | 0.000 | 2.380 |
|  | Race (Asian) | -0.523 | 0.175 | -2.987 | 0.003 | 0.593 |
|  | Race (PEER) | 0.726 | 0.192 | 3.774 | 0.000 | 2.066 |
|  | Age (50+) | -0.757 | 0.127 | -5.971 | 0.000 | 0.469 |
|  | Appointment (tenured) | -0.370 | 0.127 | -2.920 | 0.004 | 0.691 |
|  | Appointment (tenure-track) | -0.127 | 0.148 | -0.854 | 0.393 | 0.881 |
| **Academic struggle** | Gender (woman) | 0.647 | 0.094 | 6.888 | 0.000 | 1.910 |
|  | Race (Asian) | -0.650 | 0.139 | -4.658 | 0.000 | 0.522 |
|  | Race (PEER) | 0.451 | 0.181 | 2.497 | 0.013 | 1.570 |
|  | Age (50+) | -0.500 | 0.102 | -4.903 | 0.000 | 0.607 |
|  | Appointment (tenured) | -0.110 | 0.106 | -1.038 | 0.299 | 0.896 |
|  | Appointment (tenure-track) | 0.043 | 0.135 | 0.319 | 0.750 | 1.044 |
| **Disability** | Gender (woman) | 1.057 | 0.098 | 10.780 | 0.000 | 2.878 |
|  | Race (Asian) | -0.943 | 0.144 | -6.551 | 0.000 | 0.390 |
|  | Race (PEER) | 0.408 | 0.181 | 2.258 | 0.024 | 1.504 |
|  | Age (50+) | -0.392 | 0.103 | -3.801 | 0.000 | 0.676 |
|  | Appointment (tenured) | -0.084 | 0.107 | -0.781 | 0.435 | 0.920 |
|  | Appointment (tenure-track) | 0.233 | 0.137 | 1.707 | 0.088 | 1.263 |
| **CC transfer** | Gender (woman) | 0.609 | 0.097 | 6.306 | 0.000 | 1.839 |
|  | Race (Asian) | -0.434 | 0.144 | -3.006 | 0.003 | 0.648 |
|  | Race (PEER) | 0.344 | 0.187 | 1.840 | 0.066 | 1.411 |
|  | Age (50+) | -0.477 | 0.106 | -4.503 | 0.000 | 0.621 |
|  | Appointment (tenured) | -0.429 | 0.110 | -3.893 | 0.000 | 0.651 |
|  | Appointment (tenure-track) | -0.175 | 0.137 | -1.280 | 0.200 | 0.839 |
