# Supplementary material for: Women drive efforts to highlight concealable stigmatized identities in U.S. academic science and engineering
Source: PLoS One. 2023 Jul 19;18(7):e0287795. doi: 10.1371/journal.pone.0287795 (PMC10355415; doi:10.1371/journal.pone.0287795)
Supplement: S1 Appendix — (DOCX) [file pone.0287795.s006.docx]

**S1 Appendix. Copy of survey questions analyzed**

In this survey, we are interested in hearing from science and engineering faculty and instructors who regularly teach undergraduates.

I *most closely* identify as:

- A tenured faculty member in science and/or engineering
- A tenure-track faculty member in science and/or engineering
- An instructor, lecturer, teaching track, or non-tenure-track faculty member in science and/or engineering
- A graduate student or teaching assistant in science and/or engineering
- Other, please describe _____________________________________

We are trying to learn more about **potentially concealable identities** that individuals in science and engineering hold. **Potentially concealable identities** are defined as identities that others may not know you hold unless you tell them. We recognize that some identities that are visible for many individuals may be concealable, or hidden, for others. This survey will ask about some of the most common potentially concealable identities. After you select how you identify, you may be asked to what extent an identity is concealable for you personally.

With regard to gender, I most closely identify as

- Man
- Woman
- Gender-queer or non-binary
- Other, please describe __________________________________
- Decline to state

Do you identify as a member of the LGBTQ+ community?

- Yes
- No
- Decline to state

[**If yes**] Please select the word or words that best describe your identity(ies) within the LGBTQ+ community.

LGBTQ+ identities referring to sexuality and/or romantic attraction (e.g., gay, bisexual)

- Lesbian or Gay
- Bisexual
- Queer as it relates to my sexuality
- Asexual
- Pansexual
- Other, please describe ________________________________________
- Decline to state

LGBTQ+ gender identities (e.g., transgender, non-binary)

- Transgender man
- Transgender woman
- Gender-queer or gender non-binary
- Other, please describe ________________________________________
- Decline to state

I *most closely* identify as

- American Indian or Alaska Native
- Asian
- Black or African American
- Hispanic, Latino/a, or of Spanish Origin
- Native Hawaiian
- Pacific Islander
- White
- Other (including multiracial), please describe _____________
- Decline to state

I *most closely* identify as

- Someone who has or has had depression
- Someone who does not have depression
- Decline to state

I *most closely* identify as

- Someone who has or has had anxiety
- Someone who does not have anxiety
- Decline to state

I *most closely* identify as

- Growing up in a low-income household
- Growing up in a middle-income household
- Growing up in an upper-income household
- Decline to state

I *most closely* identify as

- A first-generation college student
- A non-first-generation college student (at least one of my parents or guardians has a bachelor’s degree or equivalent)
- Decline to state

I *most closely* identify as

- Someone who struggled academically in college
- Someone who did not struggle academically in college
- Decline to state

I *most closely* identify as

- Having a disability
- Not having a disability
- Decline to state

Which option most closely reflects your undergraduate college experience?

- I transferred to a 4-year institution from a 2-year college, a community college, a junior college, or a technical college
- I transferred to a 4-year institution from another 4-year institution
- I started my college career at the same 4-year institution from which I graduated
- If none of the above reflect your experience, please describe ________________________________________________
- Decline to state

Do you perceive that your **[identity]** is concealable; that is that people may not know that you identify this way unless you tell them?

- Yes, people may not know I identify this way
- No

Considering the last time you taught the science and engineering course you indicated earlier in the survey, to what extent do you reveal your **[identity]** to **undergraduates** enrolled in that science course?

- I reveal this identity to **all** undergraduates in this course (e.g., I reveal my identity to the whole class)
- I reveal this identity to **some** undergraduates in this course (e.g., during office hours)
- I do **not** reveal this identity to undergraduates in this course

***Extent of perceived stigma***

*Each participant was provided with the definition of stigmatized and then answered the following questions on a four-point scale including:*

- Extremely stigmatized
- Somewhat stigmatized
- A little stigmatized
- Not stigmatized
- I do not know what this identity is

To be **stigmatized** means to be culturally devalued, prejudiced, or negatively stereotyped due to a particular identity and is influenced by the culture of a particular context.

To what extent do you perceive that identifying as **gay or lesbian** is stigmatized in the context of academic science and engineering?

To what extent do you perceive that identifying as **bisexual** is stigmatized in the context of academic science and engineering?

To what extent do you perceive that identifying as currently or previously **having depression** is stigmatized in the context of academic science and engineering?

To what extent do you perceive that identifying as currently or previously **having anxiety** is stigmatized in the context of academic science and engineering?

To what extent do you perceive that identifying as **growing up in a low-income household** is stigmatized in the context of academic science and engineering?

To what extent do you perceive that identifying as a **first-generation college student** is stigmatized in the context of academic science and engineering?

To what extent do you perceive that identifying as **struggling academically in college** is stigmatized in the context of academic science and engineering?

To what extent do you perceive that identifying as **having a disability (e.g., learning, mental, physical, vision, hearing, etc.)** is stigmatized in the context of academic science and engineering?

To what extent do you perceive that being a **community college transfer student** is stigmatized in the context of academic science and engineering?

What is your age?

- Under 18
- 18 - 22
- 23-27
- 28-32
- 33-37
- 38-42
- 43-49
- 50-59
- 60-69
- 70+
- Decline to state
